# Proximate Factors Underpinning Receiver Responses to Deceptive False Alarm Calls in Wild Tufted Capuchin Monkeys: Is It Counterdeception?

**DOI:** 10.1002/ajp.22097

**Published:** 2012-11-26

**Authors:** Brandon C Wheeler, Kurt Hammerschmidt

**Affiliations:** 1 Cognitive Ethology Laboratory, German Primate CenterGöttingen, Germany; 2 Courant Research Centre Evolution of Social Behaviour, University of GöttingenGöttingen, Germany

**Keywords:** vocal communication, deception, skeptical responding, signal reliability, pragmatics, playback experiments

## Abstract

Previous research demonstrates that tufted capuchin monkeys use terrestrial predator alarm calls in a functionally deceptive manner to distract conspecifics when feeding on contestable resources, although the success of this tactic is limited because listeners frequently ignore these calls when given in such situations. While this decreased response rate is suggestive of a counterstrategy to deception by receivers, the proximate factors underpinning the behavior are unclear. The current study aims to test if the decreased response rate to alarm calls in competitive contexts is better explained by the perception of subtle acoustic differences between predator-elicited and deceptive false alarms, or by receivers varying their responses based on the context in which the signal is received. This was tested by first examining the acoustic structure of predator-elicited and deceptive false alarms for any potentially perceptible acoustic differences, and second by comparing the responses of capuchins to playbacks of each of predator-elicited and false alarms, played back in noncompetitive contexts. The results indicate that deceptive false alarms and predator-elicited alarms show, at best, minimal acoustic differences based on the structural features measured. Likewise, playbacks of deceptive false alarms elicited antipredator reactions at the same rate as did predator-elicited alarms, although there was a nonsignificant tendency for false alarms to be more likely to elicit escape reactions. The lack of robust acoustic differences together with the high response rate to false alarms in noncompetitive contexts suggests that the context in which the signal is received best explains receiver responses. It remains unclear, however, if listeners ascribe different meanings to the calls based on context, or if they generally ignore all signals in competitive contexts. Whether or not the decreased response rate of receivers directly stems from the deceptive use of the calls cannot be determined until these latter possibilities are rigorously tested. Am. J. Primatol. 75:715-725, 2013. © 2012 Wiley Periodicals, Inc.

## INTRODUCTION

Communication involves interactions between at least two individuals, the signaler and the receiver. The evolution of communication is thus a coevolutionary arms race between these two players, with signaling behavior evolving to influence receiver behavior in a way that preferentially benefits the signaler, while receivers evolve responses to signals that increase their own fitness [Johnstone & Grafen, 1993; Krebs & Dawkins, 1984; Seyfarth et al., 2010]. A certain degree of reliability (i.e., association with a given feature of the signaler or the environment) is necessary in order for a given signaling system to remain evolutionarily stable; in cases in which signals are highly unreliable, receivers will be selected to ignore the signal and production of the signal will become unprofitable, leading the system to collapse [Johnstone & Grafen, 1993; Maynard Smith & Harper, 2003; Scott-Phillips et al., 2012; Searcy & Nowicki, 2005].

The degree to which a given signal must be reliable in order to elicit responses will vary from signal to signal, with the costs of ignoring a reliable signal versus those associated with responding to an unreliable one being the critical factors that determine the evolutionary stable ratio [Johnstone & Grafen, 1993; Searcy & Nowicki, 2005; Wiley, 1994]. When the costs of ignoring a reliable signal are high and responding to an unreliable one are low, high rates of unreliable signaling can be evolutionarily stable, making receivers vulnerable to exploitation by signalers through the use of functionally deceptive versions of the signal (i.e., use of the signal in a context in which the receiver's regular response is maladaptive, although this does not imply an intent to deceive on the part of the signaler). Nevertheless, high rates of unreliable signaling should put selective pressures on receivers to discriminate reliability and react accordingly [Hauser, 1996]. Indeed, the Machiavellian Intelligence hypothesis argues that an ability to detect (and counter) deceptive behaviors based on cognitive mechanisms has been a major factor underpinning the evolution of large brain size and advanced cognitive abilities among primates [Byrne & Whiten, 1990, 1992].

Alarm call systems are especially vulnerable to exploitation through the use of functionally deceptive (hereafter referred to simply as “deceptive”) false alarms, as the cost of ignoring an alarm call that is actually associated with a predator detection is potentially very high [Searcy & Nowicki, 2005]. An increasing number of studies conducted in a range of taxa have demonstrated the deceptive use of predator-associated signals to elicit maladaptive antipredator reactions in receivers in competitive contexts, including feeding and mating, suggesting that the behavior may be more common than has been appreciated [Bro-Jørgensen & Pangle, 2010; Flower, 2011; Møller, 1988, 1990; Munn, 1986; Tamura, 1995; Wheeler, 2009; see also Kojima et al., 2012]. Evidence of receiver behavior, which serves to counter these deceptive alarm signals, in contrast, is rare.

Experimental field studies of tufted capuchin monkeys (*Cebus apella nigritus*, taxonomically synonymous with *Sapajus nigritus*) provide systematic evidence of both deceptive alarm calling [Wheeler, 2009] and behaviors which are at least suggestive of a counterstrategy to deception [Wheeler, 2010a]. Tufted capuchins regularly give terrestrial predator-associated calls (“hiccups”) in a range of nonpredatory contexts in which the caller would stand to gain from the antipredator reactions of listeners [Wheeler, 2010b]. For example, targets of aggression and threats frequently produce hiccups in response to such agonistic behaviors [Di Bitetti, 2001; Wheeler, 2009]. Although not conclusive evidence of deception in this case, anecdotal observations suggest that these calls may indeed be deceptive because they often distract the aggressor and, to the benefit of the caller, cause the interaction to end abruptly [Di Bitetti, 2001]. More convincing evidence for deception comes from systematic observations that demonstrate that lower ranking individuals spontaneously produce hiccups in feeding contexts in which dominant individuals can easily monopolize access to contested food resources [Wheeler, 2009]. Like aggression-elicited false alarms, these spontaneous false alarms often cause listeners to behave as if a predator is present (e.g., run higher into the canopy and out of the contested food patch) and allow the caller to obtain access to the contested resource [Wheeler, 2009].

Although deceptive false alarms elicit qualitatively similar reactions as those produced in response to predators [Wheeler, 2009], listeners are more likely to ignore those hiccups produced spontaneously during competitive feeding than they are to the same call type produced in noncompetitive contexts [Wheeler, 2010a]. The difference in responses between the two contexts results primarily from a decrease in the rate of locomotor escape reactions, while the rate of vigilance reactions is roughly equal between the two contexts [Wheeler, 2010a]. Escape reactions by listeners consume energy and allow the caller to take advantage of the listener's spatial movement; in contrast, vigilance-only reactions, while an appropriate antipredator response, are less energetically costly and do not provide the caller with any advantage in terms of opportunities to usurp the listener's spatial location. Such a decrease in escape reactions in response to alarms given in competitive contexts, relative to situations when the calls are more likely to be reliable, is suggestive of “counterdeception” (defined as a behavior which, *while not necessarily deceptive itself*, functions to reduce the success of another's deceptive behavior [Byrne & Whiten, 1990]), and may thus provide some support for the Machiavellian Intelligence hypothesis.

Understanding whether this behavior can indeed be considered counterdeception, and the degree to which it is underpinned by relatively complex cognitive mechanisms, requires understanding the proximate factors underpinning the behavior. Wheeler [2010a] suggested two nonmutually exclusive mechanisms which could underlie variation in receiver responses and support an interpretation of counterdeception. First, previous studies have shown that subtle structural variation within a given signal type can, if reliably associated with phenomena relevant to the receiver, elicit predictable variation in receiver responses [Fischer, 1998]. In the case of tufted capuchins, calls given in response to terrestrial predators are of the same general call type as those given in response to aggression and spontaneously during competitive feeding [Di Bitetti, 2001], but it is possible that there are some subtle acoustic differences between predator-elicited hiccups and deceptive false alarms. A number of studies suggest that variation within call types is sometimes associated with the caller's arousal state [reviewed in Briefer, 2012; see also Marler et al., 1992; Seyfarth & Cheney, 2003], with more averse situations tending to result in calls characterized by higher frequencies (e.g., squirrel monkeys: *Saimiri sciureus*) [Fichtel et al., 2001] and longer durations (e.g., greater false vampire bats: *Megaderma lyra*; chacma baboons: *Papio hamadryas ursinus*) [Bastian & Schmidt, 2008; Rendall, 2003]. Because predator encounters would be expected to elicit higher degrees of arousal than aggression over food from conspecifics, which would in turn be expected to lead to a higher degree of arousal than competition over food without direct aggression, then one might expect the frequency and duration parameters of hiccups given in each context to vary accordingly. However, even if such variation does exist, it does not necessarily follow that the acoustic variation will be perceived by receivers, although it would seemingly be advantageous for listeners to discriminate; playback experiments are necessary to demonstrate the ability of receivers to correctly categorize the call based on the acoustic variation [e.g., Fischer, 1998].

Second, akin to the way in which specific words in human language can acquire distinct meaning based on the broader context surrounding their use (the focus of linguistic pragmatics) [Scott-Phillips, 2010; Wheeler et al., 2011], receivers may ascribe different meanings to a signal based on broader contextual factors associated with its production [Fischer & Hammerschmidt, 2001; Rendall et al., 1999; Wheeler & Fischer, 2012; Zuberbühler, 2000]. Tufted capuchins, therefore, may be less likely to interpret hiccups produced during competitive feeding contexts as indicative of the presence of a predator than they would the same call type produced in noncompetitive contexts. At the same time, it should be noted that contextual variation in response is not necessarily indicative of contextual variation in ascribed meaning, as contextual variation also likely affects a receiver's decision in how to respond to a signal, even when its ascribed meaning is constant [e.g., Seyfarth et al., 1980; see also Fischer, in press; Wheeler & Fischer, 2012].

This study tests whether variation in response to alarm hiccups among tufted capuchins can be explained proximately by either of two nonmutually exclusive mechanisms. First, do listeners perceive acoustic differences between hiccups associated with predator encounters and those given during competition with conspecifics and react accordingly? If apparent counterdeception is indeed driven by the perception of acoustic variation, then it was predicted that the acoustic structure of predator-elicited hiccups would differ from that of deceptive false alarms produced in competitive contexts; specifically, it was expected that calls associated with predator encounters would be associated with the highest frequencies and longest durations, while spontaneous false alarms would be characterized by the lowest frequencies and shortest durations. In addition, it was predicted that playbacks (conducted in noncompetitive contexts) of calls originally recorded in the each of the two contexts would elicit antipredator reactions (i.e., vigilance or locomotor escape reactions) at different rates, with predator-elicited calls being more likely than deceptive false alarms to elicit a reaction in listeners.

Second, whether or not there are acoustic differences, receivers may vary their response based on contextual factors surrounding signal production (i.e., whether or not the call is produced in association with competitive feeding). If this is the case, then deceptive false alarms, which elicit low response rates when given during competitive feeding [Wheeler, 2010a], played back in noncompetitive contexts should elicit responses at a rate comparable to that of playbacks of predator-elicited alarms.

## METHODS

### Study Site and Subjects

Data for this study were collected in Iguazú National Park, Argentina (25°40′S, 54°30′W) from May 2005 to December 2006, May–August 2010, May–August 2011, and June–July 2012. The site sits at the southwestern edge of the South American Atlantic Forest and is characterized by a humid, subtropical climate. Further details of the study site can be found in Janson et al. [2012] and references therein. Tufted (or black) capuchins are medium-sized (2.5–3.5 kg) [Smith & Jungers, 1997], highly arboreal primates that tend to exploit the lower canopy and understory [Fragaszy et al., 2004]. Capuchins at the site are highly omnivorous, feeding primarily on fruits but spending a large proportion of time foraging for insects [Brown & Zunino, 1990]. Data were collected on three well-habituated social groups (Macuco, Rita, Gundolf) that ranged in size from 9 to 28 individuals during the study period [Janson et al., 2012]. All individuals were readily recognizable based on fur patterns and facial characteristics. Likely predators of capuchins in Iguazú include at least three species of felids (ocelots: *Leopardus pardalis*, pumas: *Puma concolor*, jaguars: *Panthera onca*) and two species of hawk-eagles (*Spizaetus ornatus* and *S. tyrannus*) [Hirsch, 2002]. Several species of venomous snakes also pose a mortal threat to capuchins at the study site [see Wheeler, 2008, 2010b]. Tufted capuchins regularly produce “hiccup” vocalizations in response to felids and snakes, while aerial predators elicit a distinct call type (“barks”) [Wheeler, 2010b]. Although not produced exclusively in predator-related contexts, hiccups regularly elicit both generalized anti-predator and terrestrial predator-specific responses, including vigilance, escape reactions, and mobbing behaviors [Wheeler, 2008, 2009, 2010a, 2010b].

This study was approved by the IACUC committee at Stony Brook University (ID nos. 2005–1448 and 2006–1448), the Animal Welfare Officer at the German Primate Center, adhered to the American Society of Primatologists principles for the ethical treatment of primates, and complied with all laws of Argentina and the EU.

### Experimental Methods

#### Acquisition of predator-elicited alarms

Simulated predator encounters were used to elicit alarm responses, which were audio recorded for comparison with alarm calls made during competitive feeding bouts. Predator encounters were simulated by presenting either a model ocelot or snake [see photos in Wheeler, 2008] (ocelot: *N* = 42; snake *N* = 7), or by playing back either a puma's vocalization (*N* = 7) [see Wheeler, 2008] or an alarm call of a white-shouldered fire-eye (*Pyriglena leucoptera*; *N* = 1), an understory anting bird whose alarm calls elicit strong alarm calling and vigilance reactions in tufted capuchins [Di Bitetti, 2001; Wheeler, unpublished data]. Additional details regarding playback methodology can be found below. Predator models and playback speakers were placed in front of the group in the direction of group movement at a distance large enough that subjects would be unable to see the observer handling the model or speaker. Snake models were always placed on the ground or a fallen tree trunk; ocelot models were normally placed on the ground, but in three cases were placed in a tree at a height of 2–5 m [see Wheeler, 2008 for additional details on simulated predator encounters]. Hiccups given in the context of visual predator models were considered to be in response to the “predator” if the subject had detected the model (i.e., ceased previous behavior and gazed in direction of model) and maintained visual contact with it when calling. Hiccups given in the context of acoustic models were considered to be in response to the playback if the calling bout began within 10 sec of the end playback. Hiccups given in response to visual and acoustic stimuli are not structurally distinguishable and were thus pooled together into a single category of “predator-elicited hiccups.”

Three recordings of hiccups used in the acoustic analysis and classified as predator-elicited were acquired under natural conditions rather than during a simulated predator encounter as described above. Two of these were detections of live venomous snakes, while the third was a response to the movements of a medium-sized mammal (a peccary: *Tayassu pecari*) in the dense forest understory. For the latter case, the similarity of the caller's behavior to situations in which predator models were used (approaching the disturbance, vigilance toward the ground) suggests that this was a case in which the caller mistakenly identified the disturbance as a potential predator and reacted accordingly.

#### Acquisition of deceptive false alarms

Audio recordings of each of aggression-elicited and spontaneous false alarms, together considered “deceptive false alarms,” were made almost exclusively in contexts in which competitive feeding was elicited through controlled provisioning experiments in which a high-value food (bananas cut into approximately 2.5-cm pieces) was presented to the subjects in approximately 1 m × 1 m wooden platforms suspended from tree branches at a height of 3–10 m [see Di Bitetti & Janson, 2001; Janson, 1996; Wheeler, 2009, 2010a]. These experiments were conducted with two of the three study groups (Macuco group in 2005, 2006, and 2010; Rita group in 2011). One to six platforms, each separated by 10–20 m from all others, were used within an experimental site, while the number of bananas distributed across the platforms at a site ranged from 2 to 30. Two to eight sites, separated by at least 200 m, were distributed throughout the home range of the study groups and were each typically baited once per day, given that the group visited the particular site. Platforms were raised as the group approached the site or, in cases in which a small portion of the group arrived separate from the others, soon after their arrival. Additional details of the platform experiments can be found in Wheeler [2009].

Hiccups given during the platform experiments were audio recorded ad libitum by an observer standing near a platform. In cases in which the caller was identified and within view of the observer in the seconds prior to the onset of the call, the call was placed into one of five categories. First, in cases in which the call was given in response to an animal, sudden noise, or movement in the understory, or if the caller ran higher in the trees or showed any other typical antiterrestrial predator behavior (e.g., branch shaking or open mouth displays and vigilance toward the ground, with the exception of cases in which individuals were searching for dropped banana pieces as described below), then the call was considered to be in response to a perceived (whether real or not) heterospecific threat, and was not used in the analysis. Second, if the call was given with 10 sec of a hiccup from a second individual, then the hiccup was considered to be a response to the first call, and was not used in the analysis. Third, if hiccups were given within 10 sec of the caller having been the victim of conspecific aggression, or if it followed within 10 sec of a bout of screams by the caller which were also an immediate response to aggression, then the hiccup was classified as aggression-elicited. Fourth, if the hiccup was given spontaneously by the caller (i.e., with no apparent eliciting stimulus and without additional antipredator behaviors) then the call was considered a spontaneous (and potentially deceptive) false alarm. While spontaneous hiccups of high amplitude (i.e., loud) are most often given by individuals just outside of platforms in which a group mate is feeding [Wheeler, 2009], hiccups of lower amplitude (but still in the range of those given in response to snakes and felids) are often given by individuals searching for banana pieces on the ground [see also Janson, 1996]. Even though this behavior by definition includes vigilance toward the ground, such calls were classified as spontaneous false alarms (potentially deceptive given that such calls would seem to decrease the likelihood that another individual obtains the dropped pieces) rather than predator-elicited if it could be reasonably assumed that the caller did not perceive a potential terrestrial predator. Specifically, to be considered a spontaneous false alarm in these cases, both the caller and the observer had to have a clear view of the ground in order to determine that no threat was present in the area where the caller was looking, the caller had to be at a height of less than 2 m above the ground and within a 5-m radius of a point directly beneath a platform (i.e., where most dropped pieces fall), and not show any additional antipredator behaviors (i.e., escape reactions and displays discussed above) immediately before or during the calling bout. That such calls were not indicative of the perceived presence of a predator is evidenced by the fact that callers almost always came down to the ground to obtain fallen fruit pieces, a behavior which would seemingly be extremely high risk in the presence of an actual predator. Further, capuchins at the study site spend approximately 3% of active time in natural contexts foraging, traveling, or playing on the ground [Wheeler, unpublished data], and such behaviors are not typically preceded or accompanied by high rates of hiccup production [Wheeler, personal observation]. Finally, if observations were insufficient to place a call into any of these four categories, then it was categorized as “unknown context” and not used in the current analysis.

One hiccup included in the acoustic analysis and classified as a spontaneous false alarm was recorded in a natural feeding context rather than during the platform experiments described above. In this case, one individual obtained a large and potentially usurpable resource (larvae from a ca. 15-cm diameter wasp nest); a second individual approached to within 2 m of the first individual and spontaneously produced hiccups while observing the first ingest the larvae. The behavior of neither individual was indicative of being stung by wasps, suggesting that this does not explain the calling behavior in this case.

#### Alarm call playback experiments

To determine whether differences in responses to alarm calls produced in competitive versus noncompetitive contexts are best explained as either the perception of structural differences of calls given in each context or the integration of contextual cues surrounding signal production, reactions of focal animals to playbacks of predator-elicited and deceptive false alarms were compared. All playbacks were conducted in noncompetitive contexts and included 12 trials of each of predator-elicited (eight distinct stimuli recorded from four adult and subadult males, one juvenile male, and three juvenile females, performed in 2005–2006; see Wheeler, 2010b] and deceptive false alarms (11 distinct stimuli recorded from five adult females, two subadult males, and four juvenile males, performed in 2010–2012). Categorization of the playback stimulus as predator-elicited, aggression-elicited, or spontaneous followed the same criteria as described above. All but two deceptive false alarms used in the playbacks were spontaneously produced hiccups that came from individuals that called in proximity to platforms (rather than those looking for banana pieces on the ground), while the other two were aggression-elicited hiccups.

Call playbacks consisted of 3–10 hiccups played over several seconds. Playbacks were conducted with a portable compact-disc player or an Apple iPod connected to a RadioShack (Fort Worth, Texas, USA; product model #277–1008) or Saul Mineroff Electronics (Elmont, New York, USA; model SME-AFS) amplified speaker hidden in vegetation at a height of 2 ± 0.5 m. Speaker volume was adjusted to mimic natural call amplitudes (approximately 80–90 dB measured at 1 m from the speaker). Playback speakers were placed approximately 10–15 m from the focal animal, and most playbacks were conducted from this distance, although the animal's distance from the speaker varied from 5 to 25 m at the moment of the playback due to the animal's movement prior to the initiation of the playback. Focal animals were video recorded for at least 20 sec prior to the initiation of the playback and up to 1 min following the playback. Videos of the experiment were used to score the focal animal's response within the first 10 sec following the initiation of the playback as either vigilance (i.e., suddenly looking toward the ground or playback speaker), escape (i.e., quick movement of 1 m or more either up or horizontally), or ignore (i.e., neither vigilance nor escape).

No more than one playback experiment was conducted per day with a given study group. Focal animals were always from the same social group as the individual whose call was used in the playback, and included 24 adult and juvenile individuals (predator-elicited alarm call playbacks: five adult females, three adult males, two juvenile females, two juvenile males; deceptive false alarm playbacks: six adult females, three adult males, and three juvenile males). Several individuals were chosen as focal animals in both types (i.e., predator-elicited and deceptive false alarms) of playbacks. In these cases, data from only the deceptive false alarm playback were included in the analysis in order to not introduce pseudoreplication, resulting from multiple data points from the same focal individual, into the analysis; the deceptive false alarm playbacks were preferentially chosen because fewer experiments of this type were conducted, and doing so allowed for a balanced sample size between the two experiment types.

### Audio Recording and Acoustic Analysis

Audio recordings were made with one of several shotgun microphone/digital audio recorder combinations. Microphones used included the Sennheiser ME-67/K6 and the Sennheiser MKH-60 P48 (Sennheiser Electronic Corp., Old Lyme, Connecticut, USA). Audio recorders included a Sony MZ-NH900 Hi-MD MiniDisc recorder (Sony Corporation of America, New York, NY, USA), a Marantz PMD-660, and a Marantz PMD-661 (Marantz America, Inc., Mahwah, New Jersey, USA). In all cases, calls were recorded in an uncompressed digital format at a sampling frequency of 44.1 kHz and 16-bit resolution. Only calls made from a distance of less than 10 m from the caller were analyzed, given that dense vegetation is known to cause considerable attenuation at larger distances [Maciej et al., 2011].

**Fig. 1:**
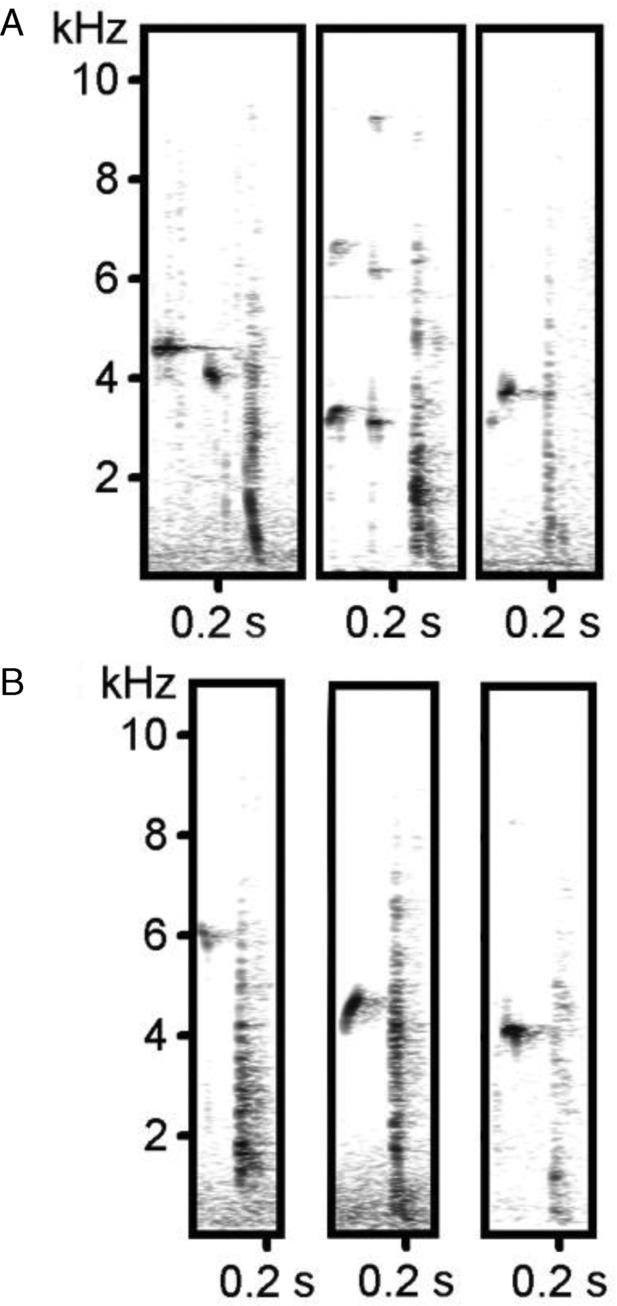
Spectrograms of, from left to right, spontaneous false alarms, aggression-elicited alarms, and predator-elicited alarms from (A) an adult female and (B) a juvenile male. Spectrogram details can be found in the “Methods.”

In all three contexts considered here (i.e., predator-elicited, aggression-elicited, and spontaneously produced calls), callers almost always produced a bout consisting of multiple hiccups, rather than a just single call. Individual calls within a bout were isolated and saved as WAV files, and then imported into the Avisoft SASLab Pro acoustic analysis package, where the sampling frequency was converted to 22 kHz at 128-bit accuracy with antialiasing filtering applied. Frequency-time spectrograms were then generated using a fast Fourier transform (1024-pt FFT, 100% frame size Hamming window, and 2.9 msec resolution). Spectrograms were visually inspected, and any that showed interference from calls from conspecifics or heterospecifics were not further considered. Remaining spectrograms were imported into LMA 2012, a custom software that extracts sets of frequency-time parameters from acoustic stimuli. Twenty-eight acoustic parameters were extracted using a custom interactive macro designed to extract two sets of parameters (Table I) from the two acoustically distinct parts of the hiccup call: the “hic-,” which encompasses a relatively narrow frequency range and typically contains one to three syllables (hics); and the “-cup,” which is a broad band noise that immediately follows the hics [Fig. 1; see also Di Bitetti, 2001; Wheeler, 2010b]. Because the automated settings in LMA were often unable to correctly determine the division between the two portions of the call, it was necessary to use an interactive macro that required the user to place a cursor at the division between the two parts of the call. Even though the two parts are typically separated by a short interval of low acoustic energy, it was often difficult to objectively determine precisely where a “hic-” stopped, due to reverberation. Thus, the cursor was always placed at the beginning of the “-cup” portion; as a result, the duration of the “hic-” portion of the call is calculated from the start time of the whole call to the start time of the “-cup” syllable. The start threshold was set at 15% and the end threshold at 20%, meaning that only those time segments after which the beginning of the call reached 15% of the mean peak amplitude (of the entire call) and before which the end of the call fell below 20% of the peak amplitude were analyzed. Because low-frequency background noise is a common issue at the study site due to its proximity to the waterfalls of the Iguazú River, it was necessary to set the cutoff frequency at 800 Hz (i.e., nothing below 800 Hz was considered in calculating the acoustic parameters of the calls). The very lowest frequencies of the “-cup” portion of some calls fell below the 800 Hz cutoff, and this may therefore increase the risk of a Type II error if calls from one context are more likely to be characterized by such low frequencies than are those from the other contexts.

**TABLE I:** Definitions of the 28 Acoustic Parameters Measured for the Current Analysis

Parameter	Definition
(A) General call parameters	
Duration [msec]	Time between onset and end of call or call section
call	Duration of the entire call
hic-	Duration of the “hic-” portion of the call, defined as the beginning of the call to the beginning of the “-cup”
-cup	Duration of the “-cup” portion of the call
(B) “hic-" parameters	
Peak frequency (PF) [Hz]	Frequency with the highest amplitude for a given time segment
Start	PF in the first time segment
End	PF in the final segment
Minimum	Lowest PF of all time segments
Maximum	Highest PF of all time segments
Mean	Mean PF across all time segments
PF max location	Location of the highest PF (ranges between 0 and 1)
PF min location	Location of the lowest PF (ranges between 0 and 1)
PF trend	Factor of the linear trend of the PF
Mean PF trend	Mean difference between the PF course and the linear trend
Maximum PF trend	Maximum difference between the PF course and the linear trend
(C) “-cup” parameters	
Distribution of frequency amplitudes (DFA) [Hz]	Describes the statistical distribution of energy in the frequency spectrum; each measurement calculated for each of the first, second, and third quartiles of this distribution
Start	The DFA in the first time segment
End	The DFA in the last time segment
Max	The maximum DFA across all time segments
Min	The minimum DFA across all time segments
Mean	The mean DFA across all time segments

(A) Parameters measured in both the “hic-“and the “-cup” portions of the calls; (B) parameters measured only in the “hic-”; (C) parameters measured only in the “-cup.”

### Data Analysis

Because nearly all bouts of calling resulted in recordings of multiple calls of similar recording quality, only one call per bout (defined as hiccups separated by at least one minute from other hiccups from the same individual) was randomly chosen to be included in analyses in order to ensure the independence of data points. To account for the high collinearity between many of the acoustic variables, a principal components analysis was used to collapse the 28 acoustic variables into seven principal components. These seven principal components together explained 85% of the total variance and primarily described: (1) the distribution of frequency amplitudes in the “-cup”; (2) the pitch of the peak frequency (PF) in the “hic”; (3) the course of the PF in the “hic”; (4) call duration; (5) distribution of frequency amplitudes and call duration; (6) the locations of minimum and maximum peak frequencies; and (7) call duration and the locations of minimum and maximum peak frequencies.

We used the seven principal components to calculate a discriminant function analysis (DFA) to test for context-specific structural features. Since DFA tends to overestimate the correct classification when data points are nonindependent, we also conducted a permuted discriminant function analysis (pDFA) using an R algorithm written by Roger Mundry, designed to determine whether the observed classification result could have been obtained by chance [Mundry & Sommer, 2007]. Because the algorithm could run only complete data sets, we reduced our data to nine subjects for which we had calls in all three conditions (i.e., predator-elicited, aggression-elicited, and spontaneous false alarms). Context was used as the test factor and subject as the control factor. Binomial tests were used to determine if the correct classification rates of the DFA and pDFA were better than those expected by chance. In addition, within-subject linear regressions were used to determine if the context in which the call was given is a significant predictor of either of the first two principal component scores (i.e., those that described the pitch-related frequency measures of each of the two parts of the call), or of the duration of the entire call. Like the pDFA, the within-subject linear regression accounts for pseudoreplication introduced by using multiple data points per individual, and also accounts for between-subject variation [Allison, 2009].

Finally, to test if playbacks (in noncompetitive contexts) of predator-elicited hiccups elicit antipredator reactions more often than do those of deceptive false alarms, a two-tailed Fisher's exact test was used to test for differences between the two treatments in, first, the likelihood that focal animals showed any antipredator reaction (i.e., a vigilance or a locomotor escape reaction vs. ignore) and, second, the likelihood of a locomotor escape reaction (i.e., escape vs. no spatial movement).

Principal component scores for each call were calculated with SPSS 17.0. The DFA was performed with SPSS 19.0, and the pDFA with R 2.11. Within-subject linear regressions were performed using Stata 10.0. Finally, the Fisher's exact tests and binomial tests were calculated using the VasserStats web utility (http://vassarstats.net/).

## RESULTS

A total of 450 hiccup calls from 163 bouts of calling were of sufficient quality for acoustic analysis. Among these, 184 calls came from 60 bouts of predator-elicited calling (from 8 adult females, 14 adult males, 9 juvenile females, and 8 juvenile males), 125 calls came from 49 bouts of spontaneously produced false alarms (from ten adult females, four adult males, three juvenile females, and five juvenile males), and 141 calls came from 54 bouts of aggression-elicited calling (from nine adult females, four adult males, three juvenile females, and six juvenile males). The regular DFA that included 163 calls (one randomly selected call per bout) indicated that 55.7% of calls were correctly classified to their eliciting context (cross-validated: 44.2%), significantly better than the chance value of 33% (binomial test of cross-validated classification: *P* = 0.003, *N* = 163). The result of the pDFA, in contrast, indicated that calls could not be assigned to their correct eliciting context better than chance (1,000 permutations: *N* = 75, *P* = 0.37; cross-validated: 0.33), with only 37% of the randomized data set being correctly assigned to their eliciting context categories, not significantly better than the chance value of 33% (binomial test: *P* = 0.67, *N* = 75).

The within-subject logistic regressions indicate that the eliciting context does not significantly predict any of the three structural parameters predicted to differ based on the caller's presumed level of arousal. Total call duration ranged from 75.4 to 327.7 msec with a mean ± SD of 183.2 ± 51.4 msec for spontaneous false alarms, 174.7 ± 49.8 msec for aggression-elicited false alarms, and 184.7 ± 44.2 msec for predator-elicited alarms. Eliciting context thus did not predict the total duration of the call (β coefficient = −3.53, *t* = −0.61, *P* = 0.542, df = 115). Likewise, there was no significant effect of context on the first principal component scores, which described the distribution of frequency amplitudes in the “-cup” portion of the calls (β coefficient = 0.14, *t* = 1.17, *P* = 0.246, df = 115). Finally, mean PF of the “hic” ranged from 3,004 to 6,913 Hz, with a mean ± SD of 4,626.6 ± 756.3 Hz for spontaneous false alarms, 4,758.0 ± 645.5 Hz for aggression-elicited false alarms, and 4,739.1 ± 655.0 Hz for predator-elicited alarms. In testing for the effect of context on the second principal component scores, which primarily reflected the five PF measurements in the “hic” portion of the calls, including the mean PF, no significant association was found (β coefficient = −0.08, *t* = 0.72, *P* = 0.475, df = 115).

**Fig. 2:**
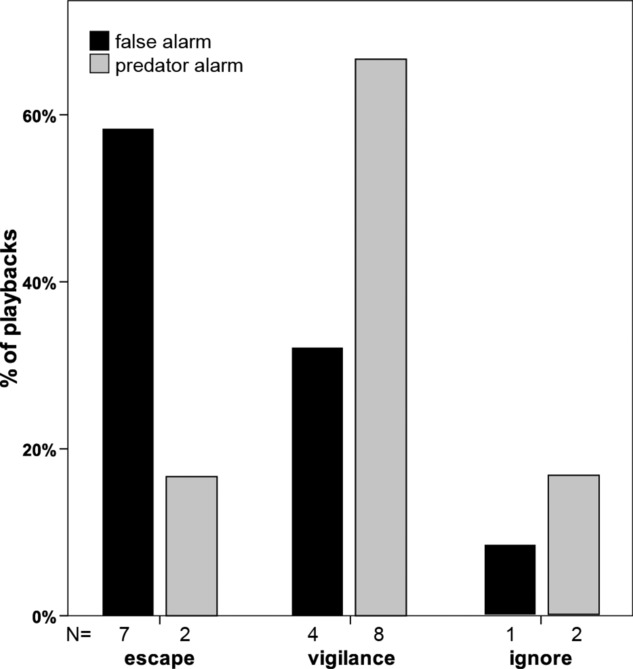
The percentage of playbacks of each of deceptive false alarms and predator-elicited alarms that elicited locomotor escape reactions, vigilance reactions, or no appropriate antipredator response. The frequency of antipredator reactions overall (including both escape and vigilance) did not differ based on the context in which the calls were originally produced. Playbacks of false alarms were somewhat more likely to elicit escape reactions, although the difference only approached significance.

Playbacks of both predator-elicited and deceptive false alarms regularly elicited antipredator reactions. In 10 of 12 cases, focal animals responded to playbacks of predator-elicited calls with an antipredator reaction (i.e., vigilance, escape, or both); similarly, 11 of 12 playbacks of deceptive false alarm calls elicited such reactions (Fig. 2). The likelihood of antipredator reactions thus did not differ based on the context in which the calls were originally produced (Fisher's exact test: *N* = 24, *P* = 1.0). In contrast, while only 2 of 12 playbacks of predator-elicited calls elicited locomotor escape reactions in focal animals, 7 of 12 deceptive false alarm calls elicited such reactions (Fig. 2). While this difference in the frequency of antipredator escape reactions approached significance (Fisher's exact test: *N* = 24, *P* = 0.089), the trend was in the opposite direction than predicted.

## DISCUSSION

The results suggest that the decreased response rate to terrestrial predator-elicited alarm calls during competitive feeding contexts among tufted capuchins [Wheeler, 2010a], when the calls are more likely to be functionally deceptive than indicative of a predator detection [Wheeler, 2009], is better explained by receivers taking contextual cues into account to determine an appropriate response than by perceiving structural differences between predator-elicited and deceptive false alarms. Although there was no significant effect of eliciting context on call parameters related to call duration, the pitch of the PF, or the distribution of frequency amplitudes, the regular DFA, based on all seven principal components and therefore encompassing more acoustic variables, was able to assign predator-elicited, aggression-elicited, and spontaneous false alarms to their correct eliciting context slightly better than expected by chance. However, the effect was rather weak and disappeared in the multivariate pDFA, which took interindividual variation into account, indicating that there is broad overlap within individuals in the acoustic structure of hiccups given in response to predators and those given in response to aggression or spontaneously in the context of strong within group contest competition for food. While it is possible that the bioacoustic methods employed were insufficient to detect potential differences between calls given in the different contexts (e.g., context-based acoustic differences may be more prominent below the 800 Hz cutoff used in the current analysis; see “Methods”), the fact that playbacks of deceptive false alarms conducted in noncompetitive contexts elicited antipredator responses at the same rate as predator-elicited alarms suggests that such structural differences do not explain why receivers tend to ignore alarm hiccups in competitive contexts. In fact, playbacks of deceptive false alarms were more likely to elicit escape reactions in noncompetitive contexts than were playbacks of calls originally given in response to predator models, although this difference only approached significance. Finally, the fact that playbacks of deceptive false alarms conducted in noncompetitive contexts were ignored much less often (16.7% of cases in the current study) than were naturally occurring hiccups in experimentally-induced competitive contexts (70.5% of cases in Wheeler, 2010a], suggests that context is likely the critical factor driving receiver responses.

Given that predator-elicited and deceptive false alarms showed, at best, minimal structural differences, it is somewhat surprising that playbacks of false alarms tended to be *more* likely to elicit escape reactions than were predator-elicited alarms, the opposite trend relative to what was expected when the eliciting context can be differentiated based on acoustic features. While the observed trends may merely be an artifact of the relatively small samples size (*N* = 24 playbacks), it is possible that there are some acoustic differences that were not detected in the current analysis. For example, while the current analysis examined only structural variation in hiccups given in different contexts, it is possible that relevant variation exists in parameters such as in intercall intervals [Rendall, 2003; Wheeler, 2010b]. If so, deceptive false alarm calls may tend to exaggerate the urgency of the supposed threat [see also Slocombe & Zuberbühler, 2007] in order to best elicit in receivers the response most beneficial to the caller (i.e., an escape reaction). It is also possible that calls given in response to predator models differ somewhat from those given to actual predators; if deceptive false alarms more closely resemble the latter, this could potentially explain why false alarm playbacks elicited more escape reactions than did those that were responses to the predator models. However, due to the rare and unpredictable nature of actual predator encounters, too few recordings from such contexts are available to test for differences between calls given to real predators and those given to models.

While the results support the interpretation that context is the driving proximate factor affecting variation in response to hiccup alarms, an additional possible explanation, not explicitly tested here, is that listeners take caller identity into account and respond less often to those individuals whose calls are more likely to be false alarms [Cheney & Seyfarth, 1988; Gouzoules et al., 1996; Hare & Atkins, 2001]. Because subordinate individuals are more likely to give deceptive false alarms than are dominants [Wheeler, 2009], it is possible that the decreased response rate in competitive contexts stems from listeners being more “skeptical” of alarms from subordinates and thus more often ignoring their alarm calls [see Gouzoules et al., 1996]. Such skeptical responding, which would seem to fit comfortably within the definition of counterdeception, could potentially explain variation in response to honest and deceptive calls in cases in which there are neither structural differences between honest and deceptive versions of the signals nor contextual cues that receivers take into account. Testing this explicitly would be best accomplished by comparing rates of responses to predator-elicited alarms from individuals of different dominance rank [Gouzoules et al., 1996]; the current data set does not allow for such an analysis, as there were too few predator-elicited alarms from dominant individuals available to use in playbacks. However, the relatively high response rate to playbacks of deceptive false alarms, all of which by definition came from individuals who produce false alarms in competitive contexts (and 10 of 12 were false alarms of lower-ranking individuals), indicates that skepticism of alarm calls based on caller identity alone is unlikely to explain the observed trends. The fact that lower ranking individuals were frequently among the first to detect the predator models [Wheeler, unpublished data] suggests that habitually ignoring alarm calls from subordinate group mates may be maladaptive. It is quite possible, however, that caller identity and broader contextual cues are simultaneously taken into account by perceivers, and that subordinates are indeed ignored more often, but only in contexts in which they are more likely to give false alarms [Gouzoules et al., 1996].

Taken together, the contention that contextual variables are important in driving receiver responses to terrestrial predator associated calls among tufted capuchins seems to be the best supported explanation for why receivers tend to ignore such calls in competitive feeding contexts. However, it remains unclear if this is indeed an example of counterdeception [sensu Byrne & Whiten, 1990]. If variation in responses were driven exclusively by perception of structural differences between honest and deceptive versions of terrestrial predator-associated alarms (i.e., if playbacks of deceptive false alarms elicited weaker or fewer antipredator reactions than did predator-elicited alarms), or by a decreased response rate to unreliable callers, then an interpretation of counterdeception would be more straightforward; such proximate factors would suggest that listener responses are driven by the different meaning they ascribe to signals with certain structural characteristics or from certain individuals [see Fischer, 1998; Gouzoules et al., 1996]. In contrast, in cases in which variation in response is best explained by context, it can be difficult to determine if the receiver is ascribing different meanings to the signal based on the context in which it is produced, or if it is ascribing a similar meaning to the signal across contexts but making a contextual decision in how to respond to the signal given that meaning [Fischer, in press; Wheeler & Fischer, 2012]. That is, receivers may invariably infer that a hiccup indicates, with a constant degree of certainty versus skepticism, that a terrestrial predator has been detected, regardless of the context in which the call is given. But even in such cases in which ascribed meaning is constant across contexts, listeners can be expected to make different decisions in how to respond to a signal based on the costs and benefits of certain responses in particular contexts. For example, vervet monkeys (*Chlorocebus aethiops*) react to eagle alarms in different ways depending on the receiver's location (in a tree vs. on the ground) at the moment the call is given, despite the similar ascribed meaning the call has across contexts [Seyfarth et al., 1980]. Likewise, capuchins may interpret all hiccups, regardless of context, as equally likely to indicate the presence of a predator, but decide to ignore hiccups during competitive feeding in order to avoid the risk of losing access to a contested resource. Considering this distinction between attribution of meaning by receivers on the one hand, and receiver decision-making given that meaning on the other, the decreased response rate can be considered counterdeceptive only if it is due to variation in attribution of meaning based on context. Variation in responses without variation in attribution of meaning would better be described as “counterdistractive” than counterdeceptive. Disentangling attribution of meaning and decision-making, however, is difficult. One possible solution to this problem is to test if other signals, such as aerial predator-associated calls, which are not used in functionally deceptive ways by tufted capuchins and appear to be consistently associated with the presence of potential aerial threats [Wheeler, 2010b], are also ignored more often during competitive feeding than in other contexts. Such conclusions regarding counterdeception therefore await additional experimental analysis.
